# Socio-Cultural Correlates of Breastfeeding Behavior Among Latina Mothers and Its Implications for Child Health

**DOI:** 10.3390/children12091109

**Published:** 2025-08-23

**Authors:** Liliana Davalos, Brisa Rodriguez Alcantar, Marissa Martinez, Christopher Johansen

**Affiliations:** Department of Social and Behavioral Health, School of Public Health, University of Nevada, Las Vegas [UNLV], Las Vegas, NV 89119, USA; rodrib36@unlv.nevada.edu (B.R.A.); martim75@unlv.nevada.edu (M.M.); christopher.johansen@unlv.edu (C.J.)

**Keywords:** breastfeeding, disparities, acculturation, Hispanics, Latinas, marital status, nativity

## Abstract

**Highlights:**

**What are the main findings?**

- Foreign-born Latina mothers had greater odds of breastfeeding than US-born Latina mothers.
- Latina mothers who were married or in a romantic relationship had greater odds of breastfeeding than mothers who were not in a relationship.

**What is the implication of the main finding?**

- Increased breastfeeding for Latino infants provides benefits that extend into childhood and are associated with decreased risk of child obesity and other conditions.
- Addressing breastfeeding disparities for Latino families can involve implementing culturally relevant interventions that emphasize cultural perspectives of breastfeeding and include extended family members.

**Abstract:**

Background: Latina mothers in the United States report lower rates of breastfeeding initiation and exclusivity than their non-Hispanic counterparts. Lower rates of breastfeeding in infancy may lead to a higher rate of excess weight in childhood and adverse health conditions. Breastfeeding disparities in Latina women have been previously correlated with socio-cultural factors such as acculturation, education, income, nativity, and marital status. This study examines whether socio-cultural factors are associated with breastfeeding behaviors in Latina mothers in Nevada. Methods: Participants were Latina mothers [*n* = 214] over 18 years of age, with a child 2–5 years old. A logistic regression was conducted to assess the associations between acculturation, age, marital status, education, and nativity with breastfeeding. Results: Mothers who were married/living with their partner had 2.3 greater odds (95% CI = 1.08, 4.73; *p*-value < 0.05) of breastfeeding than the mothers who were not in a relationship, and mothers who were born outside the US had 4 times greater odds of breastfeeding than mothers who were born in the US (95% CI = 1.178, 13.514; *p*-value < 0.05). No significant association was found for acculturation (OR = 1.13; 95% CI = 0.74, 1.7; *p*-value = > 0.05), maternal age (OR = 1.01; 95%; CI = 0.96, 1.07; *p*-value = > 0.05) and education (OR = 0.81; 95% CI = 0.37, 1.8; *p*-value = > 0.5). Conclusions: These findings suggest that a romantic partner may be supportive of breastfeeding, and foreign-born mothers may retain their cultural norm of breastfeeding. These results can be applied to develop a culturally tailored intervention to promote breastfeeding behavior.

## 1. Introduction

Breastfeeding offers various health benefits to both mothers and their child. Exclusive breastfeeding is recommended for infants during the first six months of life, after which breastfeeding can continue with complementary foods until the child is twelve months or older [1,2]. Breastfeeding provides the best nutrition for a growing infant, and contributes to improved cognitive development, protection against infections, and reduced risk of chronic conditions such as obesity, asthma, and diabetes [2,3,4,5]. The breastfeeding benefits received during infancy extend into childhood, which is crucial for children of racial minority backgrounds who have a higher rate of excess weight, and excess weight in childhood is associated with adverse health conditions later in life [6]. Benefits for the mother include decreased risk of hypertension, type 2 diabetes, breast cancer, ovarian cancer, and postpartum depression [3,5].

Despite the established benefits of exclusive breastfeeding in the first six months, there is generally low breastfeeding prevalence for infants in the United States (US), with only 24.9% of infants exclusively breastfed through six months [7]. Among breastfeeding women, low socioeconomic status, educational attainment, and the early introduction of formula have been correlated with shorter duration of exclusive breastfeeding [8,9,10]. There is a disparity in breastfeeding behavior in Latina mothers in the US, thus reducing the nutritional benefits of breastfeeding for Latino children and increasing their risk for childhood obesity. Latina women are less likely to breastfeed for the recommended six months, and one national cohort found that Latina women had 28% lower odds of breastfeeding for six months compared to their White counterparts [11]. Additionally, previous studies suggest that foreign-born Latina women living in the US had increased odds of breastfeeding compared to non-Hispanic White women [12]. This can affect health outcomes for Latino children, as breastfeeding for longer time periods has been found to prevent the development of obesity for children of Latina women recently immigrated to the US [13].

Previous studies suggest that socio-cultural factors such as acculturation, nativity, education, and marital status are correlated with breastfeeding behavior in Latina women. These socio-cultural factors influence perceived norms for breastfeeding, social support, and knowledge of breastfeeding benefits [14]. Breastfeeding behavior can be understood within the context of the Socio-Ecological Model, which examines influences at the individual, interpersonal, community, and policy levels [14]. Acculturation is the cultural and psychological changes that occur when different cultures and their members interact, typically due to migration [15]. Acculturation has been associated with other health behaviors such as less nutritious diets and insufficient physical activity [16,17]. Acculturation may influence breastfeeding rates, as highly acculturated Latina women were less likely to breastfeed than low-acculturated Latina women [18,19]. Higher educational attainment has been positively associated with breastfeeding rates, suggesting that mothers with more education are more likely to breastfeed [20,21]. Nativity is associated with breastfeeding, and a national sample found that mothers born in Mexico were nearly twice as likely to ever breastfeed than US-born White mothers [8]. Foreign-born Latinas were more likely to initiate breastfeeding and maintain this behavior for at least six months than US-born Latinas after controlling for covariates [20]. Across different ethnicities, married mothers were more likely to breastfeed than unmarried mothers, likely due to increased involvement from their partner, economic support, or emotional support in the home [22,23,24]. These socio-cultural factors influence the initiation and continuation of breastfeeding for Latina mothers, which in turn can improve health outcomes of infants and children.

The purpose of this study was to identify the socio-cultural factors that are associated with breastfeeding in a sample of Latina mothers living in Southern Nevada. Although national guidelines recommend exclusive breastfeeding for the first six months, this study focuses on initiation to better understand this disparity among Latina mothers. Breastfeeding initiation is associated with lower rates of obesity and acute illness in children [25]. This study will examine socio-cultural factors influencing breastfeeding in Latina mothers. It is hypothesized that socio-cultural factors are significantly associated with breastfeeding behaviors. Nevada has experienced rapid Latino population growth and faces persistent health disparities, making it a key state for understanding state-specific breastfeeding behaviors. Few studies have examined breastfeeding correlates at the state level among Latina mothers; none have investigated how socio-cultural factors interact to shape breastfeeding behavior in Nevada.

## 2. Materials and Methods

The data utilized in this study were derived from a cross-sectional study that primarily focused on measuring health behaviors for Latino parents and their preschool-aged children. A detailed description of the study and its measures are cited elsewhere [26]. This study involved a secondary data analysis from a convenience sample of Latina mothers from October 2022 to March 2023. Inclusion criteria were mothers who self-identify as Hispanic or Latina, at least 18 years of age, and had at least one child 2 to 5 years old. Exclusion criteria were parents who did not identify as Hispanic or Latino, whose child was not in the 2–5 age range, and individuals who were not primary caretakers of the child. The survey consisted of 41 questions that combined open-ended and multiple-choice questions and was administered in person via paper copies or through an online platform. The surveys were offered in English and Spanish, and all responses were included in the analysis. Informed consent was attained by all participants verbally and in writing. The research protocol of the study was approved by the Institutional Review Board of the University of Nevada, Las Vegas (UNLV 2021-238).

### 2.1. Measures

Acculturation. Parental acculturation was measured with Norris’ Brief Acculturation Scale for Hispanics’ (BASH) 4-item measure, which is a validated and reliable instrument [27]. The BASH is a validated acculturation measure that asks about language preference, language spoken in the home, language primarily used to think, language spoken with children, and language spoken with friends. Response options range from ‘Only Spanish’ to ‘Only English’. The acculturation measure was calculated with the mean of the 4 items. Scores ranged from 1–5, with 1 reflecting low acculturation to the US and 5 reflecting high acculturation to the US.

Breastfeeding. Participants were asked if they ever breastfed their child using a dichotomized yes/no question.

Education. Participants were asked about their highest level of education attained using a multiple-choice question from “8th grade or less” to “More than a 4-year college degree.”

Marital status. Participants were asked about their current marital status using a single multiple-choice question.

Nativity. Participants were asked if they were born in the US with a yes/no response option.

Parent Age. Participants were asked to report their current age as a whole number.

### 2.2. Data Analytic Plan

A multiple logistic regression was used to examine which socio-cultural factors were associated with breastfeeding in this sample. The dependent variable of breastfeeding was asked using a binary Yes/No question; therefore ‘no’ = 0 and ‘yes’ = 1. The acculturation score was calculated as the mean of four questions. Marital status was dichotomized, such that “married” and “living together”were coded as ‘1’ and all other responses were coded as ‘0’. The responses for education were also dichotomized such that responses indicating a high school degree or less were 0’ and responses indicating greater than a high school degree were ‘1’. Nativity was dichotomized, with ‘0’ = born in the US and ‘1’= born outside the US. The dataset met the statistical assumptions for a logistic regression, and no modifications were made to utilize this analysis. Missing values were removed from the analysis. All analyses were performed using SPSS v29 (IBM Inc., Armonk, NY, USA, 2022), and *p*-value < 0.05 was used to determine statistical significance.

## 3. Results

Descriptive characteristics of the participants are available in Table 1. A total of 256 caregivers were recruited for the study; however, in this secondary data analysis, only 214 were identified as mothers and thus were retained for this analysis. The mean age of mothers was 33.6 (SD = 6.6), and 65.9% reported ever breastfeeding their child. Most participants were born outside the US (78.5%) and primarily spoke Spanish in the home (76.6%). Approximately two-thirds (66.4%) were married or living with their partner. Nearly one-third (33.2%) of mothers had at least a high school diploma, and 43.5% of participants’ total annual household income was less than $30,000.

The results from the logistic regression model are presented in Table 2. Marital status and nativity were associated with breastfeeding behavior after controlling for acculturation, age, and education. Results suggest that mothers who were married or living with their partner had 2.3 times the odds of breastfeeding than the women who were not married/living with a partner (95% CI = 1.078, 4.734; *p*-value = 0.031). Mothers who were born outside the US had 4 times greater odds of breastfeeding than mothers who were born in the US (95% CI = 1.178, 13.514; *p*-value = 0.026). No significant association was found for acculturation (OR = 1.125; 95% CI = 0.742, 1.707; *p*-value = 0.578), maternal age (OR = 1.013; 95% CI = 0.960, 1.070; *p*-value = 0.633), and education (OR = 0.805; 95% CI = 0.366, 1.770; *p*-value = 0.589).

## 4. Discussion

The objective of this study was to examine socio-cultural factors and its association with breastfeeding behaviors among Latina mothers in Nevada. This study contributes to the increasing body of literature on breastfeeding disparities by exploring how socio-cultural factors, such as marital status and country of origin, affect breastfeeding behaviors among Latina mothers in Nevada. As the benefits of breastfeeding extend beyond infancy and are associated with decreased risk of child obesity, efforts can be made to develop culturally relevant interventions in this community [13]. As one of the first studies focused on Nevada, it provides new insights into how local cultural norms and immigrant status can influence maternal health behaviors.

Findings suggest that marital status and nativity were associated with breastfeeding behavior, which corroborates previous findings that Latina mothers born in the US had lower odds of breastfeeding [8]. This finding aligns with a previous study that found that mothers who were married or in a romantic relationship were more likely to initiate and continue breastfeeding for a longer duration, due in part to paternal support in the home and involvement with childcare [28]. Romantic partners may provide emotional and informational support to their breastfeeding partner to promote positive breastfeeding decisions. Addressing breastfeeding disparities for Latino families can not only inform interventions to increase breastfeeding rates, but it can also influence further efforts to improve Latino child health, such as healthy weight and nutrition.

Maternal nativity among Latinas has been associated with breastfeeding, as US-born Latinas initiate less and breastfeed for a shorter time than foreign-born Latinas [29]. Foreign-born Latina mothers may have increased breastfeeding rates because breastfeeding is a cultural norm in many Latin American countries and may retain these behaviors from their home countries [29,30,31]. Additionally, US-born Latina mothers are more likely to supplement breastmilk with formula, known as “las dos cosas” to address low milk supplies or experience challenges pumping in their workplace, and thus reducing the time dedicated to exclusive breastfeeding [30,32].

The results of this study diverge from previous studies that found that acculturation was associated with breastfeeding in Latina women [18]. One possible explanation for these findings may be how acculturation was measured across studies. There is no standard way to measure acculturation, and comparing the results of this study that utilized Norris’ BASH with other studies that used different scales or proxies might produce different results [33]. Additionally, most participants were foreign-born and had a low average BASH score, thus reducing the variability to detect potential differences.

Maternal age was not associated with breastfeeding. In a national sample, older mothers are more likely to breastfeed than younger mothers [34]. The sample from the current study is relatively older, with an average age of 33.6, and most participants may have prior breastfeeding experience if they had other children, which may influence breastfeeding behavior than age alone. Education was previously found to be positively correlated with breastfeeding behavior [35]. However, in the present study, 65.9% of mothers had attained a high school diploma or less. Education may not be associated with breastfeeding behavior in this study if breastfeeding is considered a cultural norm and not exclusively learned from a formal educational setting. Beyond breastfeeding, lower education levels of Latino parents have been previously found to be associated with a higher risk of child obesity [36]. This study found no association between education and breastfeeding, indicating that further research is needed to understand how maternal education influenced breastfeeding, and ultimately healthy child weight.

Understanding socio-cultural factors that influence breastfeeding behavior can be incorporated into the local adaptation of health interventions to promote breastfeeding behavior and ultimately improve the health outcomes of the child. Focusing on breastfeeding initiation and duration during infancy has indirect and downstream effects on child health, such as healthy weight. If families can adapt recommended breastfeeding behavior, they may be open to participating in other health-related interventions. Interventions to support the initiation, duration, and exclusivity of breastfeeding for the first six months include lactation support from certified counselors, interactions from peer educators, group prenatal classes, and breastfeeding-specific clinic appointments [5,37]. The finding that foreign-born Latinas have higher rates of breastfeeding can provide an opportunity to learn from other cultures where breastfeeding prevalence is high, and support women who are new immigrants to the US [30]. Interventions that consider nativity and cultural norms for some Latin American countries may involve other family members beyond the typical mother–child dyad, such as grandmothers, romantic partners, and other relatives who may be involved in raising the child [38]. If the mother is not in a romantic relationship, family members and friends could be invited to participate in educational interventions. Ultimately, effective interventions for this population should incorporate cultural norms around breastfeeding and include supportive individuals to promote recommended breastfeeding behavior. Interventions can be adapted and implemented in the Latino population in Nevada by collaborating with local Latino serving organizations, community leaders, and community clinics. Breastfeeding intervention programs should provide information in English and Spanish, utilize bilingual promotores de salud (community health workers) to connect families to services, and involve fathers and other family members to participate.

Strengths for this study include it being the first in Nevada to test whether socio-cultural variables were associated with breastfeeding behavior in Latinas. This study utilized a valid measure to assess acculturation. Additionally, this study focused on an underrepresented population that often gets overlooked, which provides an opportunity to directly address a health disparity. A limitation of this study is that it involved a secondary data analysis, and therefore, the variables available for analysis were limited and did not include important constructs such as self-efficacy, return-to-work barriers, access to healthcare or lactation support, employment status, or maternity leave. Relatedly, this study did not assess other individual-level factors that may impact breastfeeding duration such as type of delivery, illness of mother or child, medication use, and smoking. This limits the generalizability of the results to all Latina mothers living in Nevada. The survey instrument assessed breastfeeding behavior by utilizing only one measure, a yes/no question about ever breastfeeding, and did not measure exclusive breastfeeding for the recommended six months and could be subject to recall or response bias. Future research should assess exclusive breastfeeding at 6 months and continued breastfeeding at 12 months [39]. The analysis grouped all foreign-born Latina mothers into one category, when there are notable cultural differences across different Latin American countries, but due to the modest sample size, subgroup analyses were not possible.

## 5. Conclusions

This study found significant associations between marital status, nativity, and breastfeeding behavior among Latina mothers in Southern Nevada. Specifically, mothers who were married or living with a partner, as well as those born outside the US, reported greater odds of breastfeeding. These findings highlight the importance of cultural and social contexts in shaping maternal health behaviors and emphasize the need for interventions that consider family structures and the specific experiences of immigrants. Efforts to improve breastfeeding initiation and continuation seek to improve health outcomes for the children.

From a public health perspective, the results indicate the necessity of designing and implementing culturally tailored breastfeeding education programs that support both foreign-born and US-born Latinas. These programs should consider the influence of partners and extended family. Community-based efforts may benefit from integrating bilingual peer support, prenatal counseling, and culturally competent lactation services.

Future research should investigate how breastfeeding behaviors vary by country of origin, assess the duration of exclusive and continued breastfeeding, and evaluate interventions that incorporate family support networks. Understanding these socio-cultural factors is essential for reducing breastfeeding disparities and advancing equity in maternal and child health.

## Figures and Tables

**Table 1:** Descriptive Statistics of Latina mothers who participated in the survey.

Characteristics	Cases (*n* = 214)	
Parent Age (mean (SD))	33.6	(6.6)
Acculturation (mean)	2.04	(1.19)
Breastfed their child		
Yes	141	(65.9%)
No	54	(25.2%)
Missing	19	(8.9%)
Nativity		
US	37	(17.3%)
Other Country	168	(78.5%)
Missing	9	(4.2%)
Primary Language Spoken at Home		
Spanish	164	(76.6%)
English	45	(21.0%)
Missing	5	(2.3%)
Education		
≤High School Graduate	141	(65.9%)
>High School Graduate	71	(33.2%)
Missing	2	(0.9%)
Marital Status		
Married/Living Together	142	(66.4%)
Not Married	72	(33.6%)
Family Yearly income		
≤$30,000	93	(43.5%)
>$30,000	79	(36.9%)
Decline to Respond	42	(19.6%)

**Table 2:** Logistic regression models for associations between acculturation, nativity, maternal age, marital status, education, and breastfeeding among participants.

	β	Std. Error	Odds Ratio (95% Confidence Interval)	*p*-Value
(Constant)	0.161	1.047	1.175	0.878
Acculturation	0.118	0.212	1.125 (0.742–1.707)	0.578
Nativity	−1.39	0.624	0.250 (0.074–0.849)	0.026 *
Maternal age	0.013	0.028	1.013 (0.960–1.070)	0.633
Marital status	0.815	0.377	2.259 (1.078–4.734)	0.031 *
Education	−0.217	0.402	0.805 (0.366–1.770)	0.589

Dependent Variable: Breastfeeding. * Association is significant at the 0.05 level.

## Data Availability

The data presented in this study are available on request from the authors.

## Abbreviations

The following abbreviations are used in this manuscript:

UNLV	University of Nevada Las Vegas
US	United States
CI	Confidence Interval
BASH	Brief Acculturation Scale for Hispanics
SD	Standard Deviation
OR	Odds Ratio
